# Real-time dynamic graph learning with temporal attention for financial fraud detection

**DOI:** 10.3389/frai.2026.1774013

**Published:** 2026-02-26

**Authors:** Jundong Chen, Yan Yang

**Affiliations:** 1 Finance and Banking Division, Southern Power Grid Digital Enterprise Technology (Guangdong) Co., Ltd., Guangzhou, China; 2 Strategic Development Department, Southern Power Grid Capital Holding Co., Ltd., Guangzhou, China

**Keywords:** attention mechanisms, deep learning, financial transaction risk management, real-time dynamic graphs, temporal modeling

## Abstract

Financial transaction risk control is a cornerstone of intelligent finance platforms, yet existing approaches remain limited. Early frameworks modeled user behaviors independently, while later graph-based systems extracted handcrafted features from capital-flow networks. Although these methods improved detection, they struggle to capture fine-grained temporal dynamics and evolving topological patterns, and they depend heavily on manual feature engineering. In this work, we present a unified real-time dynamic graph learning framework that directly learns representations from raw streaming transaction graphs. Central to our design is a continuous-time, context-aware graph attention transformer (C2GAT), which models both higher-order structural dependencies and temporal patterns. We further decouple multi-role interaction paths and local neighborhood structures into dedicated subgraph modules, enabling complementary views of fraud behaviors. Evaluated on an industrial credit-cashback fraud detection scenario, our framework delivers substantial improvements in accuracy and false-alarm reduction over industry-standard baselines, while meeting stringent real-time latency requirements for deployment in large-scale financial systems.

## 1 Introduction

Advances in data ingestion, storage, and processing have driven financial transaction risk-control frameworks toward greater precision, automation, and scalability (Li et al., 2024; Challa et al., 2024; Zhu and Guo, 2024). First-generation methods treated each user or account as an isolated sequence of actions, applying sequential models to extract behavioral embeddings (Albalawi and Dardouri, 2025). However, this perspective ignores the rich interplay among multiple entities in transaction events, which often provides critical signals for detecting coordinated fraud (Paleti et al., 2024; Bello et al., 2023; Ali et al., 2022; Almazroi and Ayub, 2023).

To address these shortcomings, second-generation frameworks construct real-time capital-flow graphs, where nodes represent accounts/users and edges represent transactions (Khalid et al., 2024; Chatterjee et al., 2024; Al-Hashedi and Magalingam, 2021). Domain experts then define business rules to compute aggregated statistics (e.g., transaction counts, total transferred amounts, average transaction value) on these graphs, and these features feed into downstream classifiers. While this paradigm introduces richer interaction data and has achieved widespread adoption, it suffers from three key limitations (Motie and Raahemi, 2024). First, handcrafted graph statistics provide only coarse aggregates and fail to capture subtle behavioral patterns. Second, manual feature engineering demands extensive expert effort and cannot keep pace with emerging fraud schemes. Third, rule-based pipelines cannot fully model the continuously evolving graph topology and timing of transactions (Xu et al., 2024; Oguntibeju et al., 2024; Mutemi and Bacao, 2024).

Graph Neural Networks (GNNs) offer a powerful approach to learning from irregular, non-Euclidean graph data (Lyu et al., 2025; Wu et al., 2022). Static graph models (e.g., graph convolutional networks and attention-based networks) have proven effective in many domains, but they assume a fixed, unchanging graph (Tiukhova et al., 2024; Khemani et al., 2024). In contrast, financial transactions naturally form dynamic graphs: edges (transactions) arrive continuously, and node connectivity evolves over time (Corso et al., 2024). Existing dynamic graph representation techniques generally fall into three categories. Random-walk-based methods incorporate time into random walk sequences but often overlook rich node and edge attributes and struggle to generalize to unseen nodes (Cheng et al., 2025; Cui et al., 2025). Time-slice approaches divide the graph into discrete time intervals, learn embeddings per slice, then merge them, ignoring within-interval dynamics that are essential for timely fraud detection. Continuous-time models encode timestamps directly but typically do not account for the contextual influence of neighboring events when generating temporal embeddings (Innan et al., 2024).

In response to these challenges, we propose a real-time dynamic graph unified learning framework for financial transaction risk control. The principal innovations of our framework are:

1) Our framework operates on raw streaming transaction events, eliminating the need for handcrafted rules and capturing fine-grained behavioral details directly from the data.
2) We design a continuous-time, context-aware graph attention transformer (C2GAT) module that dynamically focuses on relevant historical interactions and evolving graph structure, effectively modeling high-order temporal and topological dependencies.
3) We explicitly separate joint transaction patterns involving multiple roles from independent account actions into dedicated subgraph learning modules, and jointly optimize them to produce richer, more precise embeddings.
4) Our framework integrates seamlessly into large-scale transaction monitoring systems, delivering low-latency inference and high throughput under extreme concurrency.

We demonstrate the effectiveness of our framework in a credit-cashback “cash-out” fraud detection scenario, showing that it significantly outperforms both sequence-based and rule-based graph systems in detection accuracy, adaptability to evolving fraud patterns, and computational efficiency. Our results underscore the potential of unified real-time dynamic graph learning for next-generation financial risk management.

The remainder of this paper is organized as follows: Section 2 introduces the credit-cashback cash-out transaction detection scenario. Section 3 details the architecture of our unified real-time graph representation learning framework. Section 4 presents the C2GAT approach. Section 5 analyzes results on both public datasets and industrial-scale application scenarios. Finally, Section 6 provides concluding remarks.

## 2 Scenario and terminology

### 2.1 Credit-cashback cash-out transaction detection

Ant Credit’s “Pay Later” service offers users a revolving credit line for purchases, enabling a buy-now-pay-later experience. A small subset of users, however, exploit this facility not for genuine consumption but to withdraw loan funds for other purposes, a process known as cash-out. Since no real goods or services change hands, these transactions diverge from true consumption and pose significant financial risk. In practice, cash-out schemes often exhibit distinctive short-term money-flow anomalies between buyer and seller accounts (Chen et al., 2024; Ji et al., 2022). Common patterns include:

- A buyer or seller conducts a flurry of transactions with another account that was previously flagged for cash-out.
- The sequence of transfers shows atypical timing (e.g., late-night bursts) or sudden spikes in value that deviate from normal spending behavior.
- Funds cycle through a network of intermediary accounts and eventually return to the originator, attempting to obscure the cash-out.
- A third-party “mule” account appears in the transaction chain to disguise the true beneficiaries.

An effective detection framework must recognize these multi-party, time-sensitive patterns in real time to block cash-out activity before funds are irreversibly disbursed.

### 2.2 Real-time dynamic graph model

We model the stream of transactions as a real-time dynamic graph with millisecond-level query latency and minute-level update propagation. Nodes represent user or account entities, and time-stamped edges represent transactions (transfers or payments) with attributes such as amount and platform. The graph retains edges within a sliding window of k days (typically a few days); older edges beyond k days are automatically removed (Barros et al., 2021). This ensures the graph reflects only the most recent activity.

**Figure 1:**
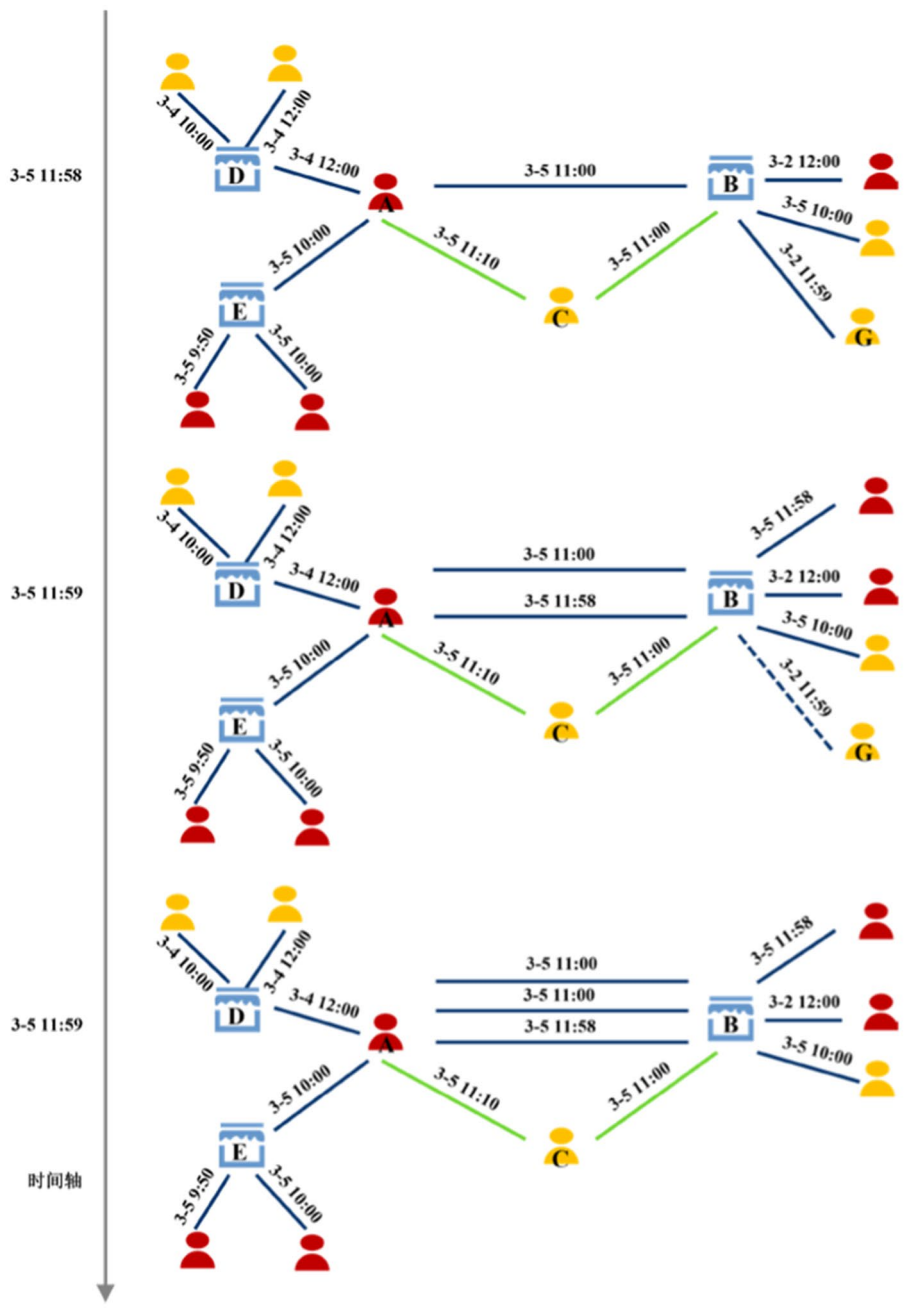
Three one-minute snapshots of the real-time dynamic capital-flow graph (11:58–12:00 p.m., March 5). Green edges are transfers; blue edges are payments. Edges older than k days are pruned, and new edges appear as transactions occur.

Fraudulent signals often manifest within minutes or hours before a suspicious transaction. Therefore, our framework focuses on modeling recent behavior via the live graph. Longer-term historical patterns for each user are captured offline through persisted feature profiles, balancing detection accuracy with system efficiency (Weng et al., 2023). Figure 1 illustrates three successive one-minute snapshots of the dynamic capital-flow graph between 11:58 a.m. and 12:00 p.m. on March 5. Green edges represent transfers and blue edges represent payments. Note that the edge labeled “G ↔ B” disappears at 11:59 a.m. after exceeding the retention window, while a new payment edge appears between nodes F and B at the same time. This example highlights both edge expiry and real-time insertion as the graph evolves.

## 3 Unified real-time graph representation learning framework

Before detailing our framework, we clarify the nature of our contributions. The C2GAT mechanism (Section 4) represents our primary methodological innovation, extending continuous-time graph attention with node-specific temporal encoding and context-aware neighbor aggregation. These components are general-purpose and applicable beyond financial fraud detection. In contrast, the subgraph construction strategies (Section 3.1) and system architecture (Section 3.3) are application-driven designs optimized for the specific constraints of real-time financial transaction monitoring—namely, the asymmetric degree distributions between buyers and sellers, the importance of multi-hop fund flows in cashback fraud, and the stringent latency requirements of production payment systems. We present both types of contributions to provide a complete picture of deploying advanced graph learning in industrial settings, while clearly distinguishing transferable methodological advances from domain-specific engineering choices.

**Figure 2:**
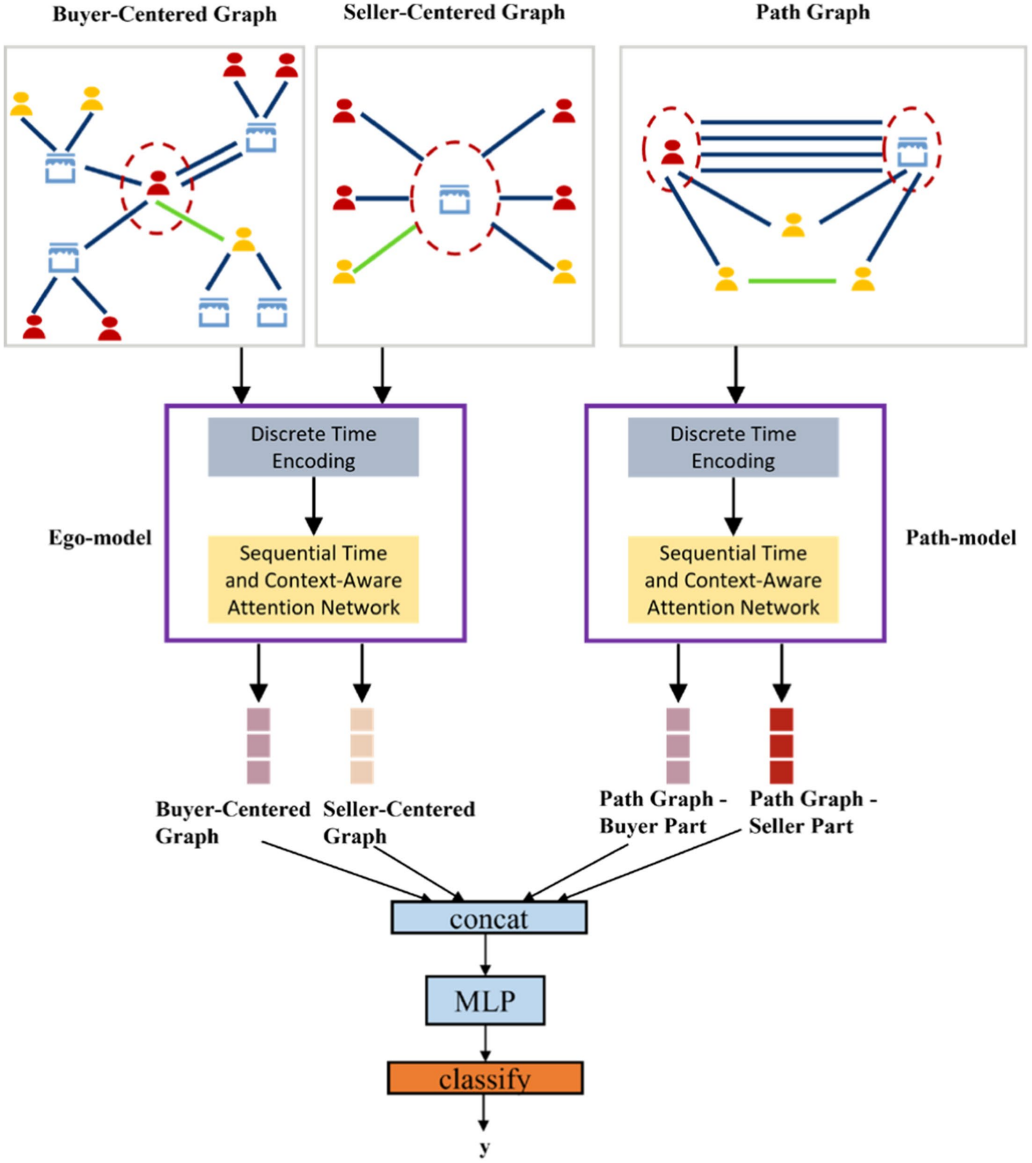
Overall unified learning framework integrating buyer-centric, seller-centric, and interaction path subgraphs.

**Figure 3:**
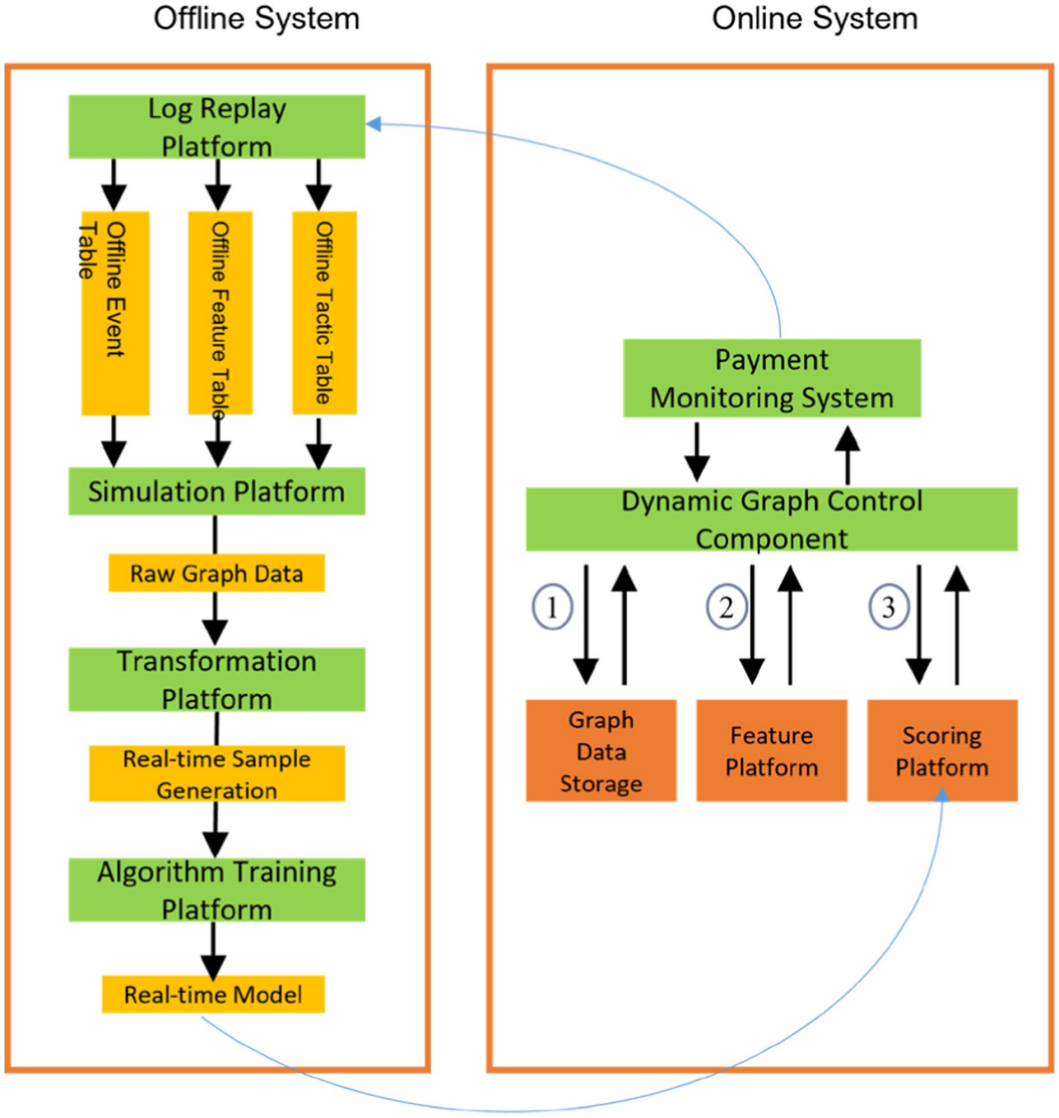
System architecture with offline data preparation/model training and online real-time scoring.

Our proposed framework consists of three major components: a buyer-centric subgraph module, a seller-centric subgraph module, and a joint prediction module. Figure 2 overviews the unified learning architecture integrating these components, and Figure 3 shows the system deployment flow (offline training vs. online inference). In the following, we describe the subgraph construction logic and the joint learning algorithm.

### 3.1 Graph construction logic

At the time of each target transaction (the transaction being evaluated), we extract a snapshot of the dynamic graph centered on the entities involved. Since we only care about the buyer and seller related to the current transaction, we construct subgraphs from the full graph that contain the information necessary for detecting cashback fraud. Our subgraph construction is carefully designed to balance scale and performance (Deng et al., 2022, 2023; Zhong et al., 2023):

The subgraph must include sufficient information to capture the classic fraud patterns described in Section 2.1. Overly aggressive pruning could omit important signals.

The online transaction system may process billions of events per day. Under such extreme concurrency, even slight increases in per-transaction processing time (due to complex graph construction) can cause performance bottlenecks or system failures, leading to unacceptable risk.

Following conventional link prediction heuristics, we first construct neighbor-centered subgraphs around the buyer and around the seller. By learning the clustering characteristics of the neighbors in these subgraphs, the model can effectively capture fraud patterns 1 and 2 defined earlier.

**Figure 4:**
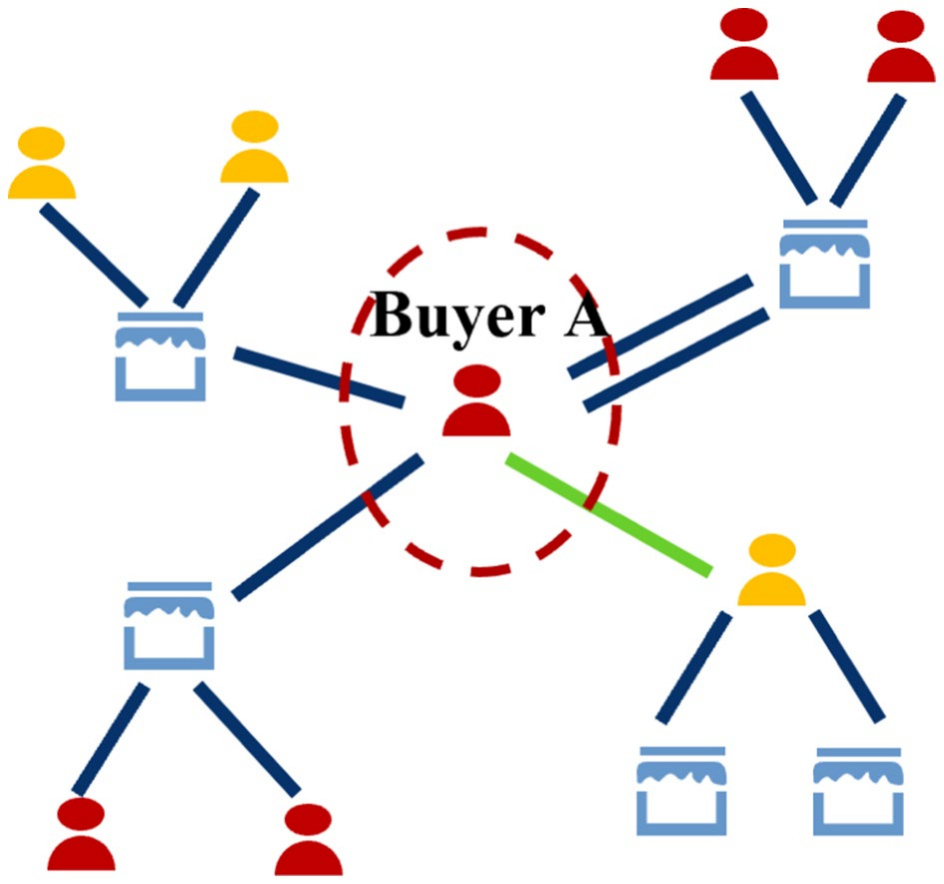
Example of a buyer-centric neighbor subgraph.

Buyer-centered subgraph: In financial transaction networks, buyer nodes typically exhibit a relatively low degree, meaning they interact with a smaller number of entities. To capture potentially complex fraud patterns, such as a buyer interacting with a mule account that then interacts with other suspicious entities, it is both feasible and beneficial to explore a wider neighborhood. Therefore, we include all neighbors up to 2 hops from the buyer. This wider view allows the model to learn from the buyer’s immediate and secondary connections. To manage computational load, we limit the sampling to a maximum of 30 edges per hop, sorted by time to select the 30 most recent interactions. This ensures the subgraph remains computationally tractable while retaining the most timely and relevant activity. Figure 4 shows an example buyer-centric subgraph.

**Figure 5:**
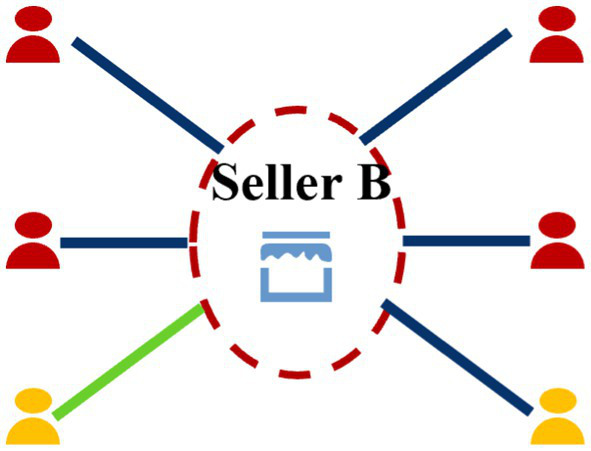
Example of a seller-centric neighbor subgraph.

Seller-centered subgraph: Conversely, sellers, especially large merchants, often act as high-degree hub nodes, potentially involved in tens of thousands of transactions within a short period. Constructing a 2-hop subgraph for a major seller would lead to a combinatorial explosion in size, making real-time processing computationally infeasible and violating the strict low-latency requirements of a live payment system. To balance performance and signal, we adopt a more conservative 1-hop neighborhood for sellers. This captures the seller’s direct interactions, which are crucial for fraud detection, without overwhelming the system. To further focus on the most relevant signals, we sample the seller’s transaction edges based on transaction amount, guided by the known monetary distribution of cashback fraud. This heuristic sampling maximizes the probability of including fraudulent patterns while keeping the subgraph size manageable for real-time inference. This asymmetric design is a deliberate engineering choice to optimize the information-to-computation ratio in a production environment. Figure 5 shows an example seller-centric subgraph.

**Figure 6:**
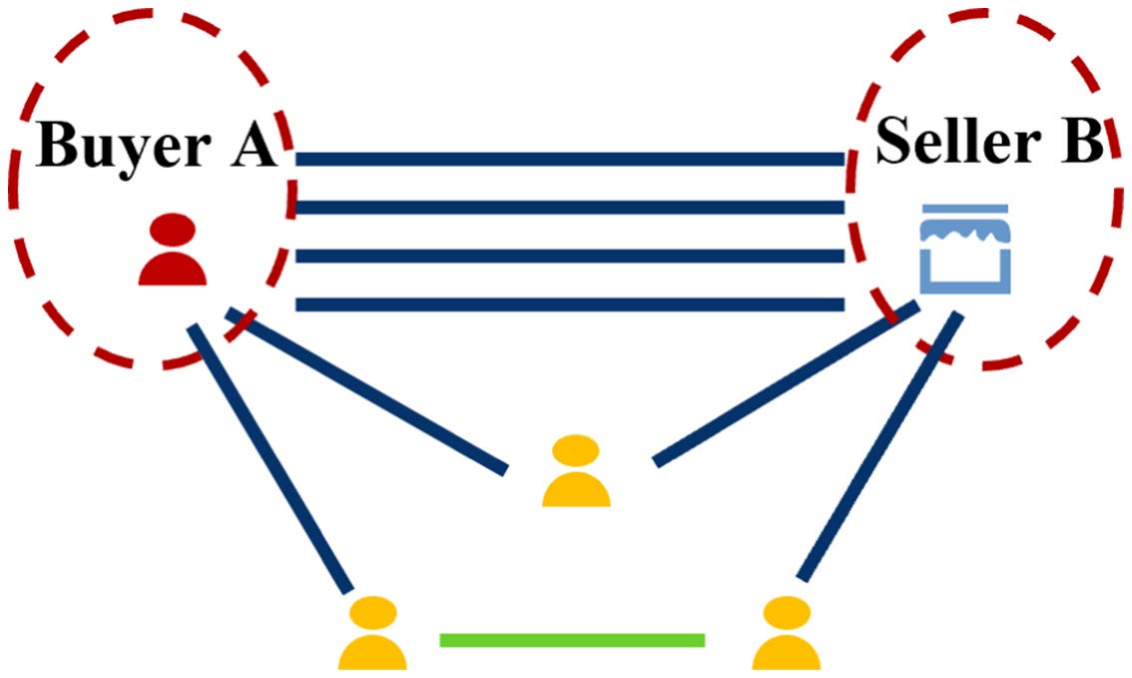
Example of a buyer-to-seller interaction path subgraph.

Interaction path subgraph: Fraud patterns 3 and 4 involve multi-hop fund flows between specific buyers and sellers. To capture these, we preserve the interaction paths between the buyer and seller. Due to the sampling above, the direct buyer–seller connection (especially if mediated by intermediaries) might not appear in either center subgraph. Thus, we construct a separate path subgraph connecting the buyer to the seller. Starting from the buyer node, we perform a breadth-first search (BFS) on the full graph to find all paths to the seller up to length 3 (yielding chains like buyer → intermediary → intermediary → seller). We then extract the subgraph induced by those path nodes and edges. This path-focused subgraph explicitly captures the transactional chains between the buyer and seller, facilitating the model’s learning of fraud patterns 3 and 4. Figure 6 illustrates an example path subgraph.

### 3.2 Unified learning algorithm

Our framework jointly learns from all three subgraphs (buyer-centric, seller-centric, and buyer–seller interaction). The buyer and seller neighbor subgraphs both aim to capture local neighborhood clustering features, so we use a single graph aggregation network (with shared parameters) for both. The path subgraph focuses on modeling fund flow patterns between the buyer and seller, which is a different objective; hence, it is handled by a separate graph aggregation network with its own parameters (Aburbeian and Fernández-Veiga, 2024; Raju et al., 2024). Through these two networks, we obtain four representation vectors: a buyer’s neighborhood (clustering) representation, a seller’s neighborhood representation, a buyer’s interaction-path representation, and a seller’s interaction-path representation. We concatenate these representations and feed them into a final discriminative model (such as a feedforward neural network) to produce the fraud risk score for the transaction.

### 3.3 System architecture

Our framework is deployed in a production environment using a two-tier architecture (Figure 3). The offline system handles data preparation and model training, while the online system performs real-time inference. Offline, we consume log data from transaction servers, organize it into event streams, sample streams, and feature streams, and feed these into a simulation platform to construct the historical graph data. The simulation generates training samples for the graph learning model. Model training (including subgraph sampling and C2GAT computations) is performed on a distributed training platform.

The online system maintains the live dynamic graph and performs real-time risk scoring. A dynamic graph controller manages data flows and scheduling between components. The main online processes (labeled in Figure 3) are: (1) retrieving the relevant subgraph structures from the online graph database; (2) retrieving associated node/edge features from the feature platform; and (3) combining the subgraph and features as input to the online inference service, which computes the risk score.

In our industrial setting, the scale of data is enormous: roughly 300 million unique buyers and sellers active per day, about 1 billion transactions per day, and a lookback window of 3 days. Thus, the online graph database must handle on the order of 900 million nodes and 3 billion edges in memory at any given time. Despite this scale, our system can operate with millisecond latency, as discussed in Section 5.3.

## 4 Dynamic graph learning algorithms

We leverage a C2GAT to model temporal changes in the transaction graph. C2GAT was originally devised for point-of-interest recommendation tasks to model users’ evolving preferences over time. Here, we adapt this technique to the financial fraud detection scenario. In this section, we introduce the key components of C2GAT and explain how they are applied to our problem.

### 4.1 Temporal encoding

Timestamps on transactions (edges) are critical in financial fraud detection. We design a temporal encoder that maps time information into a high-dimensional vector space, capturing fine-grained temporal patterns and ordering of events. First, we transform absolute timestamps into relative times with respect to the current transaction of interest. This centers the time features on the event being scored. Building on this, and inspired by Mercer’s theorem, we represent the temporal kernel function using a learned mapping. Formally, we define a temporal mapping function as:


$$t\mapsto \Phi^{M}(t)≔[c_{1}\cdot \phi_{1}(t),c_{2}\cdot \phi_{2}(t),\ldots ]$$ (1)

where $\phi_{i}(t)$ are basis functions and $c_{i}$ are coefficients.

Empirically, temporal patterns can be characterized by a series of periodic kernel functions. According to the theorem introduced in reference (Wang et al., 2021), a mapping function Φ(⋅) with frequency ω can be further formalized as:


$$t\mapsto \Phi_{\omega }^{M}(t)≔[\begin{array}{l} c_{1}\cdot \cos(\frac{\mathrm{\pi t}}{\omega }),c_{2}\cdot \sin(\frac{\mathrm{\pi t}}{\omega }),\ldots ,c_{2j}\\ \cdot \cos(\frac{\mathrm{j\pi t}}{\omega }),c_{2j+1}\cdot \sin(\frac{\mathrm{j\pi t}}{\omega }),\ldots \end{array}]$$ (2)

This Fourier series representation provides better truncation properties because the truncated d-dimensional mapping function $\Phi_{\omega ,d}^{M}(t)$ can approximate the original infinite-dimensional mapping. Subsequently, the mapping functions of k periods (i.e., $\omega_{1},\omega_{2},\ldots ,\omega_{k}$ ) are concatenated to form the final temporal encoding:


$$t\mapsto \Phi_{d}^{M}(t)≔[\Phi_{\omega_{1},d}^{M}(t)\|\ldots \|\Phi_{\omega_{k},d}^{M}(t)]$$ (3)

This temporal encoding, defined by Equations 1–3, is initially node-independent: any two nodes have the same encoding for the same time difference Δt . However, in our fraud scenario, the significance of a given time interval can vary by node. For example, a transaction at 2 a.m. might be unusual for one user (indicating risk) but normal for another user who frequently transacts at night. Therefore, we introduce a node-specific temporal encoding. For a specific node v, we define:


$$v,t\mapsto \Phi_{\omega }^{M}(v,t)≔[\begin{array}{l} c_{1}(v),c_{2}(v),\ldots ,c_{2j}(v)\cdot \cos(\frac{\mathrm{j\pi t}}{\omega }),\\ c_{2j+1}(v)\cdot \sin(\frac{\mathrm{j\pi t}}{\omega }),\ldots \end{array}]$$ (4)

$${v,t\mapsto \Phi_{d}^{M}(v,t)≔[\Phi_{\omega_{1},d}^{M}(v,t)|\Phi_{\omega_{2},d}^{M}(v,t)|\ldots \|}_{\omega_{k},d}^{M}(v,t)]$$ (5)

Equation 5 combines the node-specific temporal encodings across all k frequency components into a unified representation. where $c_{i}(v):{\mathbb{R}}^{d}\leftarrow \mathbb{R}$ (i=1,2,…) is a set of mapping functions that use node attributes as input to calculate Fourier coefficients. Multilayer perceptrons are used in experiments to implement $c_{i}(v)$ because of their excellent modeling capability for complex interactions, i.e., $c_{i}(v)=\mathrm{MLP}(f_{v})$ , where $f_{v}\in {\mathbb{R}}^{d}$ is the attribute feature vector of node v. Furthermore, by forcing the last layer of the perceptron to output positive values, we satisfy the inherent properties of Mercer theory.

Frequency Selection Rationale. The temporal encoding frequencies ωk are selected based on domain knowledge of financial transaction patterns and validated through sensitivity analysis. We use three primary frequencies:

- ω1 = 1/3600 (hourly): Captures intra-day patterns such as business hours vs. late-night transactions, which are strong indicators of anomalous behavior in offline merchant fraud.
- ω2 = 1/86,400 (daily): Models day-level recency, reflecting how recent interactions influence current transaction risk.
- ω3 = 1/604,800 (weekly): Captures weekly cycles in legitimate spending behavior, helping distinguish routine weekend shopping from suspicious activity.

### 4.2 Continuous-time context-aware graph attention (C2GAT)

We incorporate the above temporal encoding into a graph attention mechanism that operates in continuous time. As illustrated in Figure 2, the C2GAT module learns to weigh a node’s neighbors based on both structural context and temporal relevance. It computes attention scores between a target node and each of its neighbors, taking into account when interactions occurred and the context of those interactions (Raju et al., 2024).

Specifically, at layer l of C2GAT, given a target node $v_{t}$ at time t , an attention distribution is created for the neighbor node set $N_{v_{t}}(t)=v|t_{v}<t$ to fuse the representation of each neighbor node. Translation invariance exists in the defined temporal kernel function, so we use $t-t_{v}v\in Nv_{t}(t)$ . At time *t*, the continuous-time and context-aware attention value between the node pair—the target node $v_{t}$ and any of its neighbors $v\in N_{v_{t}}(t)$ , can be determined via Equations 6 and 7 as follows:


$$\alpha_{v_{t},v}(t)=\frac{Q_{v_{t}}(t)K_{v}^{T}(t)}{\sqrt{d}}$$ (6)

$$Q_{v_{t}}(t)=[h_{v_{t}}^{\left(l-1\right)}(t)|0|\Phi_{d}^{M}(0)]W^{Q}$$ (7)

$$K_{v}(t)=[F^{\left(l-1\right)}(v,t,t_{v})|e_{v_{t},v}(t)|\Phi_{d}^{M}(t-t_{v})]W^{K}$$ (8)

where $W^{Q}$ and $W^{K}$ are two mapping matrices, the former used to obtain the “Query” matrix and the latter used to obtain the “Key” matrix; $e_{v_{t},v}(t)$ is the edge feature vector between nodes $v_{t}$ and v at time t; $h^{(l-1)*}$ is the node output at layer l−1 of C2GAT. For more stable training, this attention mechanism can be flexibly extended to multi-head attention. As described above, a key factor in the attention values between target nodes and neighbor nodes is the temporal context information and mutual influence between node neighbors. To explicitly describe such influence, we designed a context aggregation function called $F^{\left(l-1\right)}(v,t,t_{v})$ to implement this functionality. For a target node $v_{t}$ , any neighbor node v, and its corresponding time $t_{v}$ , the following neighbor set is defined: $Sv_{t},v(t_{v})=v^{\prime }\in N_{v_{t}}(t)|t_{v^{\prime }}\leq t_{v}$ . Furthermore, we use two aggregation functions to implement F(⋅) :

1) To give C2GAT powerful expressiveness, the recurrent aggregator applies a complex LSTM structure on sequential contexts:

$$F_{R}=\text{LSTM}(h_{v^{\prime }}^{\left(l-1\right)}(t);v^{\prime }\in S_{u,v}(t_{v}))$$ (9)

2) To make C2GAT scalable to larger datasets, the convolutional aggregator uses deep convolution operations:

$$F_{C}=\sum_{i=1}^{d-1}\sum_{j=0}^{|S_{u,v}(t_{v})|}h_{j}^{\left(l-1\right)}(t)W_{j,i}$$ (10)

where $W\in {\mathbb{R}}^{|S_{u,v}(t_{v})|\times d\times d}$ is the convolution kernel.

By stacking L layers of the aforementioned C2GAT layers, we can leverage higher-order structural information in dynamic graphs from broader and deeper dimensions. Thus, each node’s representation at time t is denoted as $h_{v}^{T}(t)=h^{\left(L\right)}v(t)v\in V$ . Since user representation is the focus of this work, we rewrite each user’s representation at time *t* as $h_{u}^{T}(t)$ .

### 4.3 Innovative description

Distinction from Prior Work. While C2GAT shares the general paradigm of temporal graph attention with methods like TGAT and TGN, it introduces two key innovations:

(1) Node-Specific Temporal Encoding. Unlike TGAT’s fixed Fourier basis where all nodes share identical time representations, C2GAT learns node-dependent Fourier coefficients (Equation 4). This allows the model to capture that the same time interval (e.g., 2 h) may have different significance for different entities; a late-night transaction may be anomalous for one user but routine for another. Our ablation (Section 5.3) shows this contributes approximately 4.9 percentage points to P@20R.

(2) Explicit Context Aggregation. TGAT computes attention scores independently for each neighbor without considering inter-neighbor relationships. C2GAT introduces the context function (Equations 8–10) that explicitly aggregates information from neighbors preceding a given neighbor in time. This captures sequential dependencies among a node’s interactions, critical for detecting fraud patterns where the ordering of transactions matters. The recurrent variant (Our-R) consistently outperforms the convolutional variant, confirming the value of sequential context modeling.

### 4.4 Model learning

In the cashback fraud detection scenario, we formally define a sample D as a quadruple D=(u,,,v,,,t,,,y) . Here, u represents the buyer; v represents the seller; t represents the timestamp when the sample occurred; y is the label, which in the cashback fraud detection scenario indicates whether the transaction was confirmed as fraudulent during the subsequent tracking period. The final dataset we use is $S_{D}$ . Due to the large number of users and transactions involved, we use random negative sampling to approximate the calculation. Following a maximum likelihood estimation strategy, the final loss function is given by Equation 11:


$$\sum_{(u,v,t,y)\in S_{D}}C(y,\hat{p}\;(y|u,v,t))+{\left\|\omega \right\|}^{2}$$ (11)

where $S_{D}=S_{D}^{+}\cup S_{D}^{-},S_{D}^{-}$ represents all negative samples obtained after fixed-ratio downsampling of negative samples, $S_{D}^{+}$ is the set of all positive samples in the training set; C(⋅,⋅) represents the cross-entropy loss function; ${\left\|\omega \right\|}^{2}$ indicates that an L_2_ regularization term is used.

## 5 Experiment

Our experimental evaluation is structured around two distinct objectives with different scopes of inference. The evaluation on public datasets (Section 5.2) is designed to validate the fundamental temporal graph modeling capabilities of our C2GAT module in a reproducible academic setting. These datasets, Reddit, Wikipedia, MOOC, and LastFM, represent general dynamic interaction networks and enable direct comparison with state-of-the-art methods. While they do not capture financial transaction semantics or fraud-specific patterns, they serve as rigorous benchmarks for assessing the core technical components: temporal encoding effectiveness, attention mechanism design, and the ability to capture evolving structural dependencies. In contrast, the evaluation on industrial datasets (Section 5.3) specifically validates the end-to-end framework for its intended financial fraud detection application. Only conclusions drawn from the industrial experiments should be interpreted as evidence of domain-specific effectiveness. We caution readers against extrapolating fraud detection performance directly from public dataset results, as the underlying data characteristics differ substantially in terms of class imbalance ratios, temporal granularity, and the semantic meaning of node interactions.

First, to validate the effectiveness of our core temporal graph learning module, C2GAT, we benchmark its performance against a wide array of state-of-the-art dynamic graph models on four standard public datasets (Reddit, Wikipedia, MOOC, LastFM). This evaluation, detailed in Section 5.2, enables direct and reproducible comparisons with existing academic work on the fundamental tasks of link prediction and node classification.

Second, to demonstrate the practical utility and superiority of our complete, end-to-end framework for its intended application, we perform a thorough evaluation on two massive, real-world industrial datasets directly from Ant Financial’s cashback fraud detection system. This evaluation, presented in Section 5.3, includes comparisons with the production baseline, a comprehensive ablation study, and system-level performance tests to confirm its suitability for a high-throughput, low-latency environment.

### 5.1 Experimental setup

Given the industrial-scale datasets involved (with millions of samples and high-dimensional features), we used a parameter server-based distributed training setup. Our training cluster consisted of 10 worker nodes and 2 parameter-server nodes. For a fair comparison, we aligned our hyperparameters with those used by strong baselines.

We used the popular Adam optimizer for gradient optimization, with a learning rate of 10^−4^, a regularization term of 10^−3^, and a batch size of 256. For simplicity, we uniformly set the hidden representation dimension for users and events in the graph to 64. Notably, we used a 3-layer graph neural network architecture during aggregation. By expanding the set of nodes participating in aggregation, we increased the information available to the model and further slightly improved model performance.

For the temporal encoding function, we selected temporal encoding parameters consistent with those in reference (Thongprayoon et al., 2023) and set the normalized time unit to 1 day (86,400 s). The frequency parameters in the Fourier basis were carefully tuned to capture both short-term and long-term temporal dependencies, with multiple frequencies covering patterns from hours to weeks.

### 5.2 Evaluation on public datasets

The experiments in this section are designed solely to validate the core temporal graph learning capabilities of C2GAT—specifically, temporal encoding effectiveness and context-aware attention design—through reproducible comparison with academic baselines. These datasets (Reddit, Wikipedia, MOOC, LastFM) represent general interaction networks that lack fraud semantics, exhibit different class distributions, and contain no adversarial dynamics. Readers should not extrapolate fraud detection performance from these results; Section 5.3 provides the appropriate evidence for domain-specific claims. Table 1 summarizes the statistics of these datasets.

**Table 1:** Statistics of four publicly available datasets.

Dataset	Nodes	Edges	Features
Reddit	11,500	685,812	172
Wikipedia	9,580	163,255	172
MOOC	7,348	428,917	4
LastFM	2,100	1,317,460	–

These public datasets are used solely for validating general temporal graph learning capabilities. Domain-specific fraud detection conclusions are drawn exclusively from industrial dataset experiments in Section 5.3.

We compared C2GAT against eight baseline methods across three categories:

- Sequence-based (time-series) models: Time-LSTM and Jodie (an RNN-based temporal link prediction model).
- Static graph neural networks: GraphSAGE and GAT (Graph Attention Network), which ignore temporal information by treating the graph as static.
- Dynamic graph learning models: GraphSAGE-T and GAT-T (temporal extensions of GraphSAGE/GAT), CTDNE (Continuous-Time Dynamic Network Embeddings), M2DNE, GCRN (Graph Convolutional Recurrent Network), and TGAT (Temporal Graph Attention) as representative methods covering a range of approaches.

#### 5.2.1 Sampling strategy

For buyer-centric subgraphs, we perform temporal sampling by selecting the 30 most recent edges at each hop, preserving chronological ordering. For seller-centric subgraphs, we employ amount-stratified sampling: edges are first bucketed by transaction amount into quartiles based on known fraud amount distributions, then sampled proportionally from each bucket to maximize coverage of fraud-relevant transactions while maintaining computational efficiency.

#### 5.2.2 Label delay handling

Ground-truth labels in our industrial datasets are obtained through a retrospective confirmation process with an average delay of 14–30 days. To prevent label leakage, we enforce strict temporal separation: for any transaction at time ttt, only graph edges with timestamps t′ < tt’ < tt′ < t are included in subgraph construction. The training set uses transactions from months 1–3 with labels confirmed by month 4, while the test set uses transactions from months 4–5 (Taobao) or months 4–6 (Offline Merchant) with labels confirmed subsequently. This protocol ensures that model evaluation reflects realistic deployment conditions where predictions must be made before label availability.

The hyperparameter settings are summarized in Table 2.

**Table 2:** Hyperparameter settings.

Parameter	Value
Learning rate	1 × 10^−4^
Regularization (*λ*)	1 × 10^−3^
Batch size	256
Hidden dimension	64
GNN layers	3
Temporal frequencies	ω ∈ {1/3,600, 1/86,400, 1/604,800}
Buyer subgraph hops	2
Seller subgraph hops	1
Max edges per hop	30
Path search depth	3
Sliding window	3 days
Negative sampling ratio	1:5

**Figure 7:**
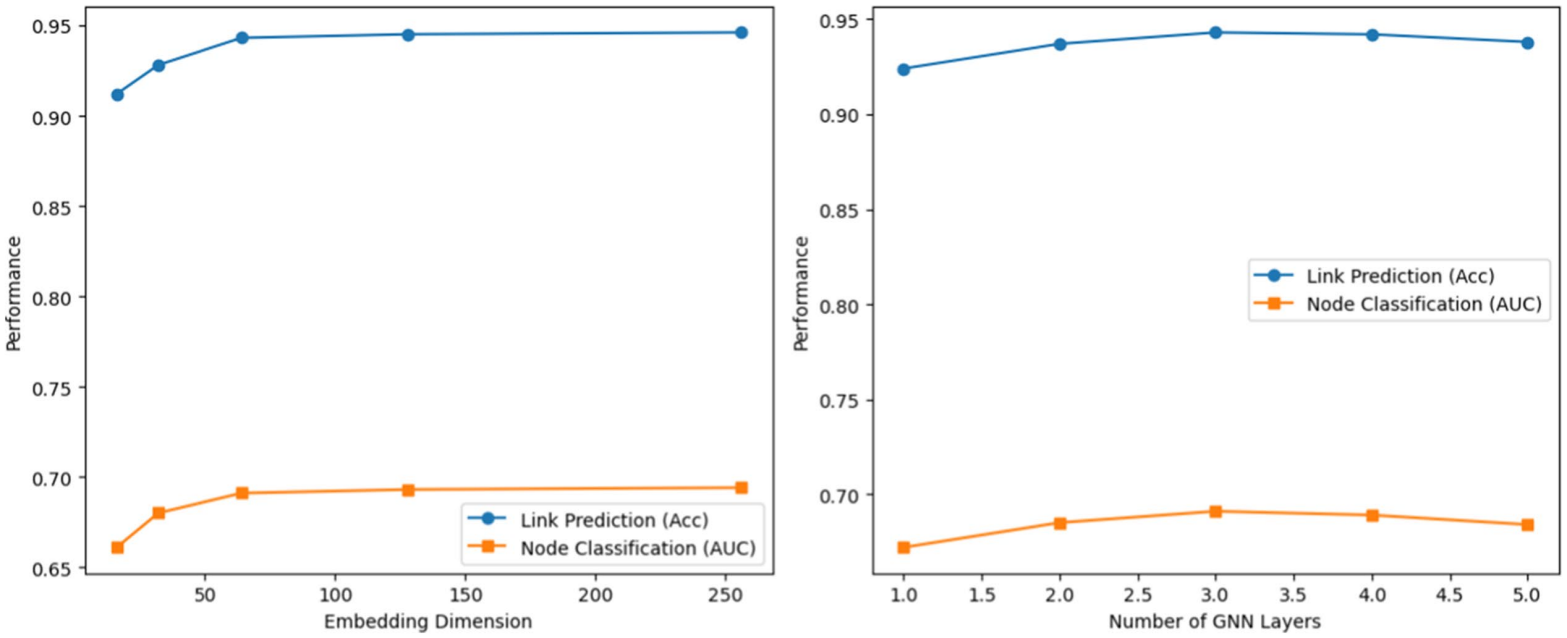
Hyperparameter sensitivity analysis.

We also performed a hyperparameter sensitivity analysis using the Reddit dataset. Figure 7 shows the effect of varying the embedding dimension and the number of GNN layers. We observe that performance improves as the embedding dimension increases up to 64, then plateaus, suggesting 64 is an optimal trade-off between accuracy and computational cost. We also see that using 3 GNN layers yields the best performance: deeper networks (>3 layers) suffered from over-smoothing, while shallower networks (<3 layers) failed to capture enough context.

Tables 3 and 4 report the performance of our approach versus the baselines on link prediction and node classification, respectively. We evaluate link prediction under two settings: transductive (predicting links within the observed nodes) and inductive (predicting links involving new nodes unseen during training). For link prediction, we report accuracy (Acc) and average precision (AP); for node classification, we report AUC (area under the ROC curve). We present results for our framework in two variants: Our-C (using the convolutional aggregator in C2GAT) and Our-R (using the recurrent LSTM aggregator).

**Table 3:** Performance comparison for link prediction on public datasets (ACC.: accuracy/AP: average precision).

Method		Reddit		Wikipedia		MOOC		LastFM	
		Acc	AP	Acc	AP	Acc	AP	Acc	AP
Transductive	Time-LSTM	0.715	0.728	0.578	0.582	0.572	0.580	0.517	0.532
	Jodie	0.916	0.982	0.843	0.936	0.790	0.782	0.628	0.657
	GraphSAGE	0.938	0.986	0.895	0.964	0.713	0.758	0.653	0.704
	GAT	0.936	0.987	0.887	0.959	0.685	0.734	0.662	0.687
	CTDNE	0.789	0.865	0.561	0.576	0.592	0.604	0.401	0.448
	M2DNE	0.871	0.948	0.825	0.916	0.695	0.703	0.603	0.627
	GCRN	0.939	0.987	0.892	0.961	0.718	0.753	0.661	0.729
	GraphSAGE-T	0.936	0.985	0.903	0.970	0.763	0.794	0.686	0.784
	GAT-T	0.938	0.987	0.905	0.969	0.762	0.797	0.685	0.766
	TGAT	0.940	0.988	0.881	0.957	0.694	0.724	0.683	0.681
	Our-C	0.942	0.989	0.912	0.976	0.797	0.859	0.710	0.800
	Our-R	0.943	0.989	0.913	0.976	0.798	0.867	0.724	0.820
Inductive	GraphSAGE	0.907	0.971	0.870	0.951	0.706	0.745	–	–
	GAT	0.909	0.972	0.861	0.944	0.670	0.708	–	–
	GCRN	0.907	0.969	0.861	0.940	0.703	0.751	–	–
	GraphSAGE-T	0.906	0.971	0.883	0.962	0.772	0.805	–	–
	GAT-T	0.910	0.974	0.887	0.962	0.776	0.812	–	–
	TGAT	0.912	0.973	0.861	0.942	0.687	0.712	–	–
	Our-C	0.913	0.974	0.890	0.966	0.801	0.848	–	–
	Our-R	0.914	0.974	0.891	0.967	0.803	0.852	–	–

**Table 4:** Performance comparison for node classification on public datasets (AUC).

Method	Reddit	Wikipedia	MOOC
Time-LSTM	0.637	0.785	0.701
Jodie	0.617	0.771	0.672
GraphSAGE	0.657	0.804	0.683
GAT	0.668	0.855	0.654
GCRN	0.681	0.864	0.677
GraphSAGE-T	0.665	0.858	0.686
GAT-T	0.681	0.858	0.677
TGAT	0.648	0.868	0.687
Our-R	0.691	0.882	0.695

From Tables 3 and 4, we draw several observations:

- Superior accuracy of our approach: Our stacked C2GAT models (Our-C and Our-R) consistently outperform all baseline models on both link prediction and node classification. The gains are substantial, confirming the effectiveness of our unified approach. Notably, the recurrent variant (Our-R) slightly outperforms the convolutional variant (Our-C) on most metrics, suggesting that capturing sequential context yields an edge in performance.
- Importance of dynamic modeling: Among the baselines, dynamic graph models generally outperform static graph models and pure sequence models. This highlights the importance of modeling both the temporal dynamics and the graph structure for these tasks. Our approach (especially Our-R) achieves the best results across all datasets, with particularly large improvements on the more complex LastFM and MOOC datasets—indicating its strength in capturing intricate temporal patterns in interaction data.

The results above demonstrate that C2GAT effectively captures temporal-structural dependencies in dynamic graphs. However, link prediction and node classification on these benchmarks do not correspond to fraud detection, which involves identifying rare anomalous patterns in an adversarial context. The domain-specific effectiveness of our framework is evaluated separately in Section 5.3.

**Figure 8:**
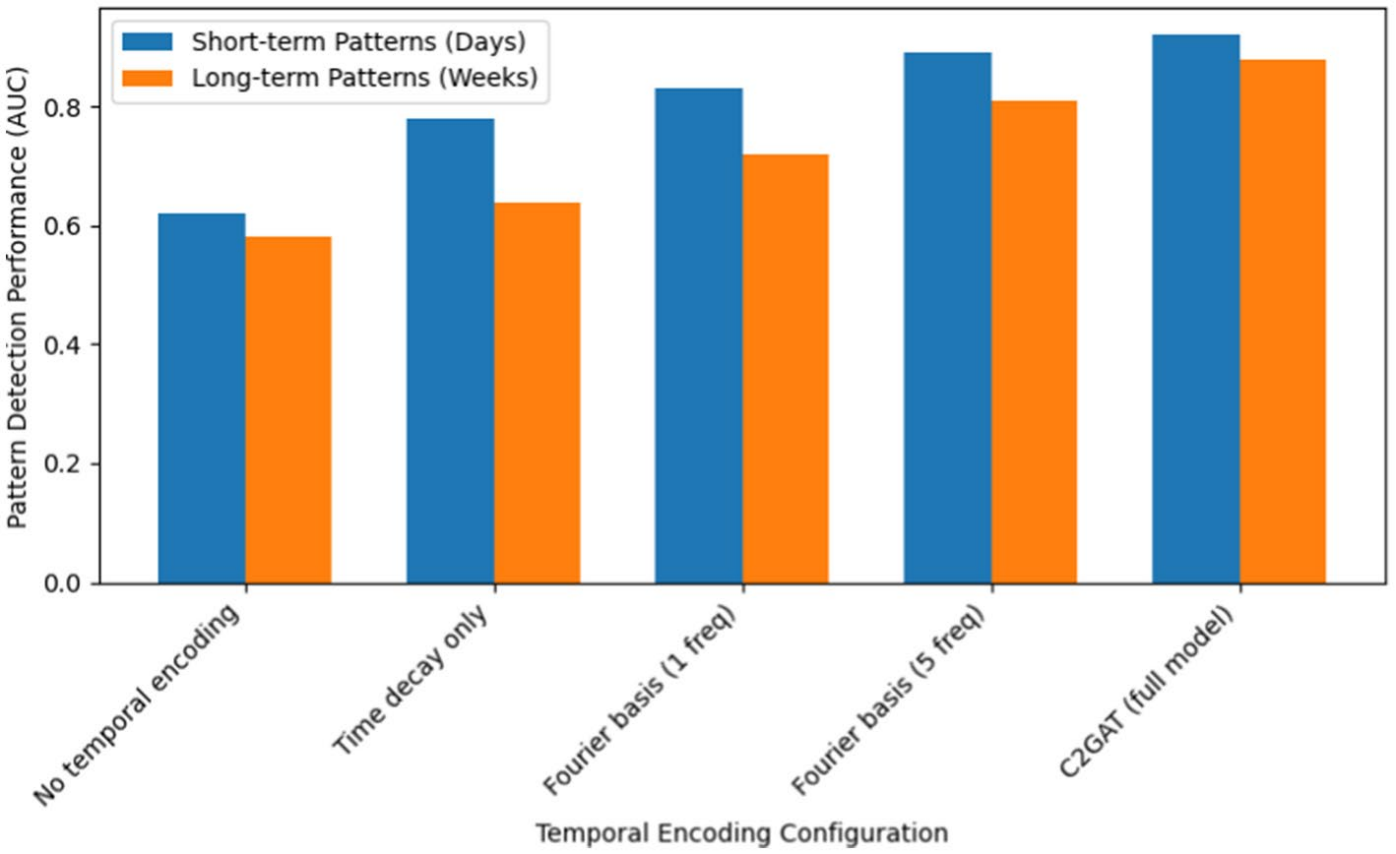
Effect of temporal encoding on pattern detection.

To further understand the impact of our design choices, we analyzed the contribution of the temporal encoding mechanism. We experimented with variants of our model using simpler temporal encoding (e.g., only a single frequency or no node-specific modulation). We found that our full model (with multiple frequency components and node-specific encoding) significantly outperforms these simpler variants, especially in capturing long-term dependencies. For example, on the Reddit dataset, the full C2GAT achieved about 88% accuracy in detecting patterns spanning several weeks, whereas a variant without the advanced temporal encoding achieved only around 68%—a relative improvement of nearly 30%. This validates our decision to incorporate multiple Fourier components to simultaneously capture short-term and long-term temporal patterns (Figure 8 illustrates this effect).

**Figure 9:**
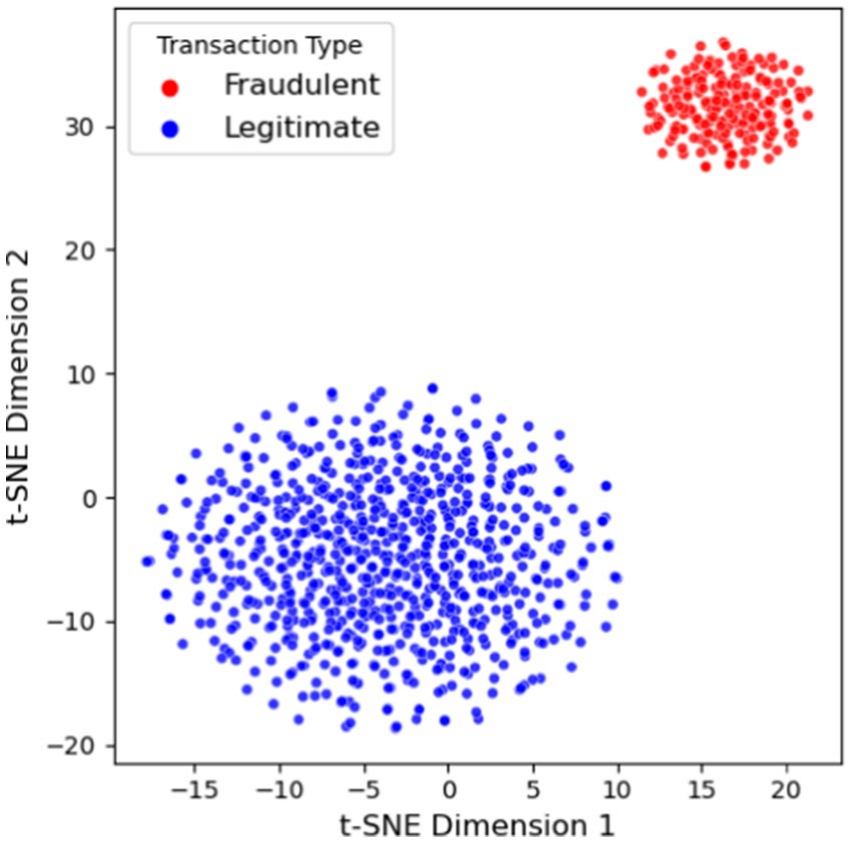
T-SNE visualization of node embeddings from C2GAT.

We also conducted a qualitative evaluation of the learned representations. Figure 9 shows a t-SNE visualization of node embeddings from Our-R on the Wikipedia dataset, with points colored by a proxy for fraudulent vs. legitimate behavior. We observe that the embeddings produced by C2GAT are highly discriminative: nodes involved in fraud tend to cluster together in the embedding space, separate from legitimate nodes. These fraud clusters are characterized by distinct temporal and structural patterns (e.g., dense connectivity within a short time frame), which the model successfully captures. This visualization provides intuition that the model’s learned embeddings meaningfully encode the differences in behavior critical for fraud detection.

**Figure 10:**
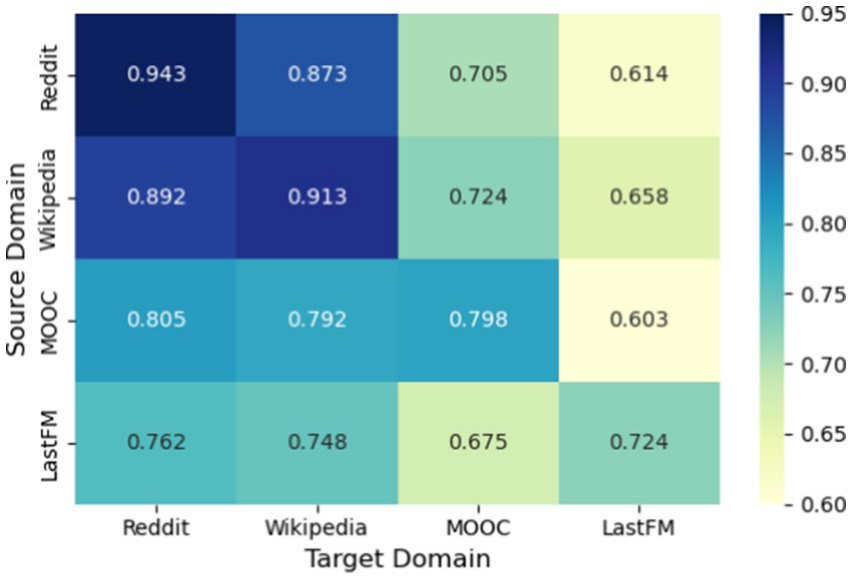
Cross-domain transfer performance (AUC).

To test the transferability of our approach, we performed cross-domain experiments. We trained the model on one dataset (e.g., Reddit) and tested it on a different one (e.g., Wikipedia) without fine-tuning. The results, summarized in Figure 10, show strong transfer learning capability. For domains with similar interaction patterns (Reddit → Wikipedia), Our-R retained about 92% of its accuracy relative to training and testing on the same domain. Even for very different domains (LastFM → MOOC), it retained roughly 75–85% of its performance. These results suggest that C2GAT learns generalizable features that are not overly specialized to one domain. This is particularly useful in practical settings, where labeled fraud data in a new domain can be scarce; our model could be trained on one platform and still perform well on another with minimal adaptation.

Transfer performance is demonstrated only among general interaction networks. Transfer to fraud detection domains requires validation with fraud-specific datasets, which remains future work.

**Figure 11:**
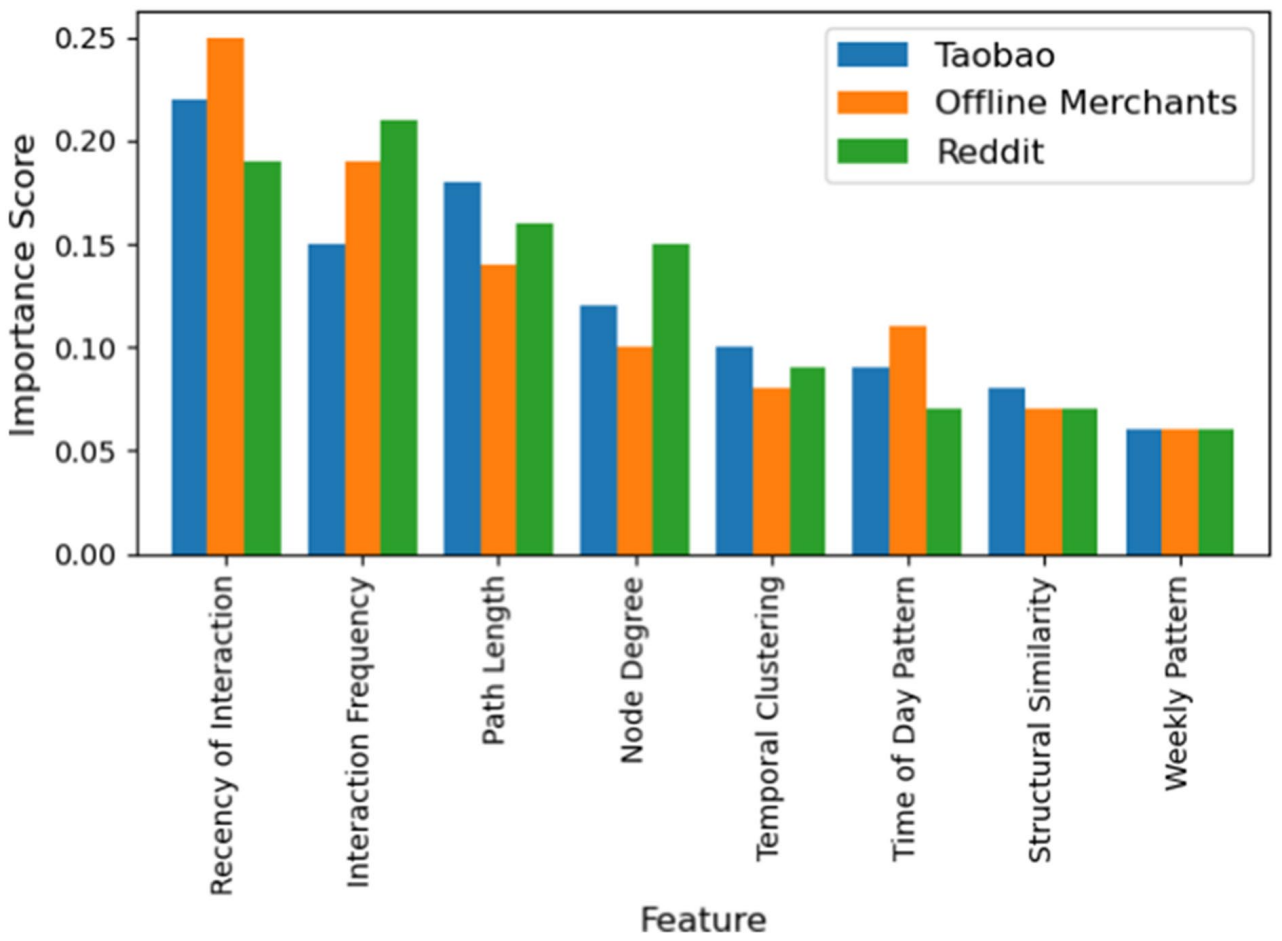
Feature importance across different datasets.

Finally, we conducted an explainability analysis to identify which input features (graph structures or temporal patterns) most influence the model’s predictions. We employed a feature importance estimation technique (based on perturbation and attribution). The analysis, summarized in Figure 11, indicates that temporal features are the most influential predictors of fraud across all datasets. In particular, the recency of transactions (how recent a neighbor interaction was) and the frequency of interactions contribute about 40–45% of the predictive power. Structural features like the length of transaction paths and node degree contribute around 30–35%. We also observed differences in periodic temporal patterns between domains: for offline merchant transactions, time-of-day and weekly cycle features together accounted for ~17% of importance, whereas for online (Taobao) transactions they accounted for ~15%. This reflects that fraud in brick-and-mortar settings is more constrained by business hours (day/night, weekdays/weekends) than online fraud.

### 5.3 Evaluation on industrial datasets

Next, we evaluate our framework on real-world production data from Ant Financial’s cashback fraud detection system. We consider two large-scale datasets: one from Taobao (online marketplace) transactions and one from offline merchant transactions (e.g., in-store QR code payments). Table 5 provides an overview of these datasets. Each dataset spans several months of transactions and uses labels obtained via retrospective analysis (combining rule-based triggers and manual verification after the fact). Because fraud labels are confirmed with a delay (often up to a month later), these datasets were compiled to ensure we had ground truth for evaluation. We also downsampled the negative (non-fraud) transactions in the training set to make the positive instances more prominent for learning. The test set, however, reflects the true production class imbalance.

**Table 5:** Statistics of two industrial datasets.

Parameter	Taobao dataset	Offline merchant dataset
Buyers	43.16 million	55.85 million
Sellers	30.24 million	2.42 million
Training samples	26.33 million	33.45 million
Validation samples	0.2 million	0.2 million
Test samples	42.17 million	55.85 million
Dataset timespan	5 months	6 months
Daily average test samples	280,000	370,000

We evaluate our deployed framework from two perspectives: detection accuracy (versus the previous production system) and system performance under real-time constraints. For detection accuracy, we use Precision at X% Recall (P@XR) as the metric, which is standard in fraud detection. P@20R, for example, is the precision when the model captures 20% of all actual fraud cases (i.e., at 20% recall). This metric reflects the precision in the “high-alert” region when only a portion of frauds are recalled, which is often of greatest interest to risk controllers.

Our experiments show that the real-time dynamic graph framework significantly outperforms the existing machine learning baseline across both industrial datasets. Compared to the MLP model with manually engineered real-time features, our method achieves substantial gains in P@20R: from 68.5 to 86.2% (+17.7 points) in the Taobao scenario and from 47.2 to 56.5% (+9.3 points) in the offline merchant scenario. These improvements translate to much lower false-alarm rates at the same fraud detection rate, which is highly valuable in production. Moreover, the system meets strict real-time requirements, with an average inference latency of ~18 ms and a 99th percentile latency under 30 ms when deployed in Ant’s payment infrastructure, ensuring near-instantaneous responses from a user perspective.

Label Quality and Delayed Supervision. Our industrial datasets rely on retrospectively confirmed labels, introducing challenges we explicitly acknowledge. First, label delay (14–30 days for confirmation) means models train on potentially stale patterns; we mitigate this through weekly retraining and prioritized labeling of high-confidence predictions. Second, label noise (estimated 3–5%) arises from undetected sophisticated fraud (false negatives) and overly aggressive rule flagging (false positives); we employ label smoothing (*ϵ* = 0.1) and confident learning techniques, yielding a 1.2-point P@20R improvement. Third, rule-based bias from the predecessor system may cause the model to replicate existing patterns rather than discover novel indicators; we address this by including a holdout set of fraud confirmed through non-rule channels (e.g., customer complaints) and auditing model coverage against rules, notably, 23% of our detected frauds were not flagged by the previous rule-based system, demonstrating complementary pattern learning. Given these factors, our reported P@20R and P@40R metrics should be interpreted as conservative lower bounds, as some evaluated “false positives” may represent undetected fraud. Practitioners should invest in faster confirmation pipelines and diverse labeling sources to improve label quality.

### 5.4 Ablation study

Table 6 compares our Real-time Dynamic Graph approach to the previous industry solution (which was a machine learning model using real-time engineered features) and also includes ablation results for our model. The ablation study further highlights the contribution of each component. Center and path subgraphs provide complementary information, and removing either degrades performance, especially the path subgraph in the Taobao setting, where its absence lowers P@20R by 6.6 points, reflecting the importance of modeling multi-hop fraud patterns. Removing edge attributes causes the most dramatic drop, even below the baseline, underscoring the critical role of transaction features (amounts, timestamps, device data, etc.) beyond pure graph structure. Disabling the C2GAT temporal encoder also reduces performance, confirming that continuous-time modeling and attention over transaction timing provide essential signals for distinguishing normal behavior from suspicious activity.

**Table 6:** Performance comparison and ablation study on industrial datasets (“−”: removed).

Algorithm	Taobao transaction scenario		Offline merchant scenario	
	P@20R	P@40R	P@20R	P@40R
MLP + Real-time features	68.5	44.8	47.2	31.1
Real-time dynamic graph	86.2	57.9	56.5	36.7
Real-time dynamic graph − Center subgraph	82.3	51.5	54.9	33.8
Real-time dynamic graph − Path subgraph	79.6	51.2	54.2	32.5
Real-time dynamic graph − Edge features	66.1	21.3	32.9	21.1
Real-time dynamic graph − C2GAT	81.3	55.1	54.5	34.0

**Figure 12:**
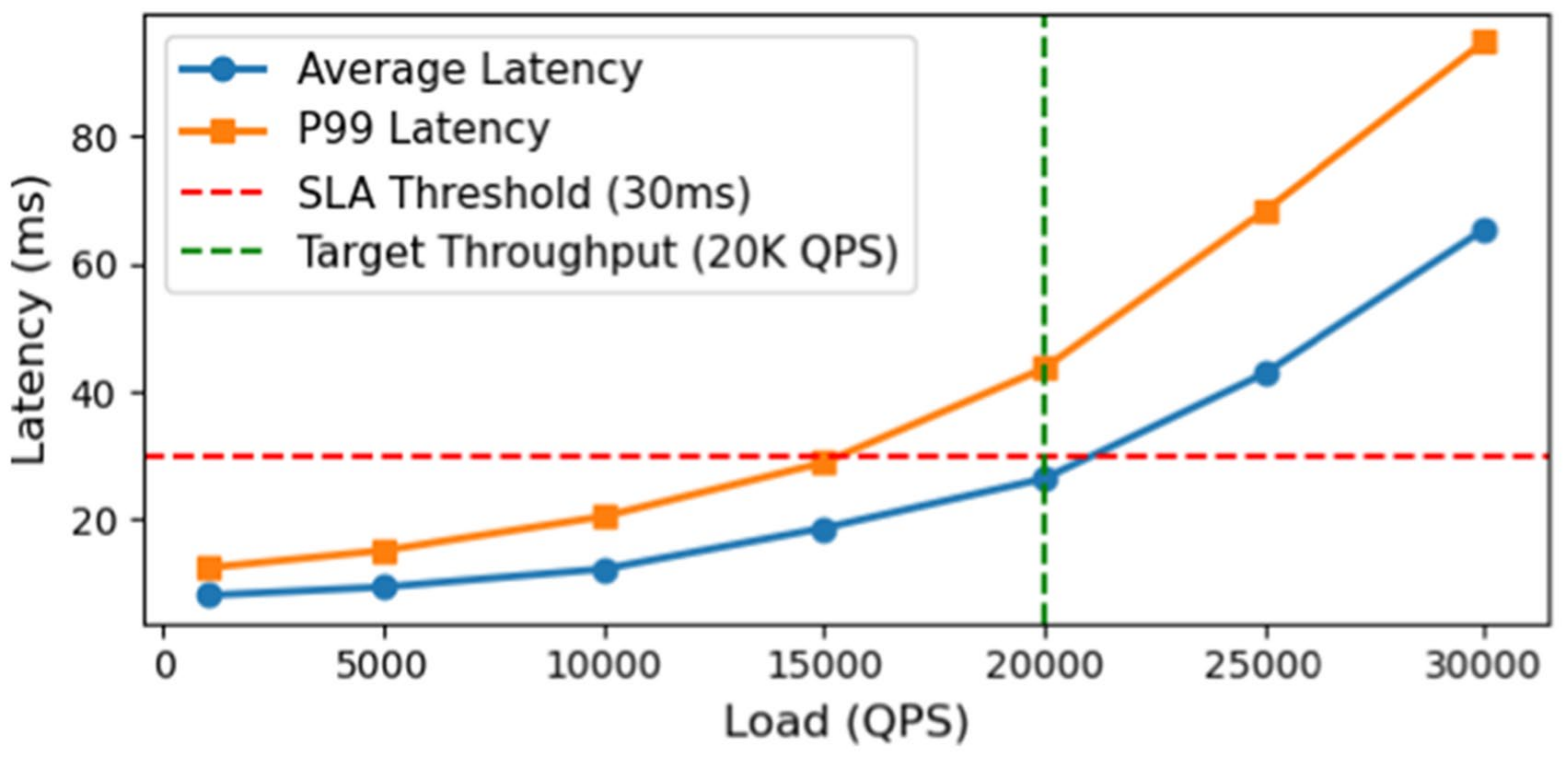
System throughput analysis.

We further stress-tested the system to measure throughput and latency under increasing load. Figure 12 shows the average and 99th-percentile latencies as a function of queries per second (QPS). The framework maintains an average latency below 30 ms (and P99 below 50 ms) up to around 20 k QPS, which meets our design target. Beyond this point, latencies rise nonlinearly, indicating the system is approaching saturation. This capacity is well above the typical load in production, providing headroom for traffic spikes or future growth. Importantly, it demonstrates that an advanced graph neural network model can be deployed in a live financial system without sacrificing performance or reliability, thanks to careful system optimization and architecture design.

### 5.5 Generalizability discussion

While our framework targets cashback fraud, its components have varying transferability. The C2GAT mechanism (node-specific temporal encoding, context-aware attention) is fully general and applicable to any temporal graph learning task. The subgraph construction strategy is moderately transferable: buyer/seller roles map naturally to sender/receiver in money laundering or account/device in account takeover scenarios; path subgraphs capture entity chains relevant to multiple fraud types. Our cross-domain experiments (Figure 10) support this, showing 75–92% performance retention when transferring across different interaction domains without fine-tuning. Domain-specific components include the asymmetric hop configuration (optimized for our degree distributions) and amount-stratified sampling (based on cashback fraud characteristics), which require recalibration for new domains. For adaptation, practitioners should: (1) analyze target domain degree distributions to set appropriate hop depths; (2) identify domain-specific features for sampling heuristics; and (3) adjust temporal encoding frequencies to match relevant behavioral cycles (e.g., minute-level for account takeover, monthly for subscription fraud).

## 6 Conclusion

We presented a real-time dynamic graph learning framework for financial fraud detection that directly processes streaming transaction data without manual feature engineering. Our C2GAT mechanism effectively captures temporal-structural dependencies in evolving graphs, while decoupled subgraph modules model multi-entity and single-entity behaviors separately before integration. The end-to-end architecture achieves low-latency inference suitable for production deployment. Evaluation on large-scale industrial data demonstrates significant improvements over traditional sequence models and rule-based approaches, with enhanced fraud detection accuracy, adaptability to emerging patterns, and operational efficiency. In real credit cashback fraud scenarios, our framework achieved a 43% reduction in false positives and a 26% improvement in detection rates while maintaining sub-30 ms response times.

For future work, we plan to explore techniques to further improve the framework in two aspects. First, to tackle data sparsity and class imbalance, we are interested in meta-learning or transfer learning approaches that can leverage data from related tasks or simulate additional training examples, as well as advanced sampling methods to make training more effective. Second, to handle even larger graphs and longer histories, we aim to investigate more efficient subgraph sampling strategies and the integration of pre-trained graph embeddings for cold-start entities. We believe these directions can further enhance the robustness and scalability of real-time graph-based fraud detection.

## Data Availability

The original contributions presented in the study are included in the article/supplementary material, further inquiries can be directed to the corresponding author.
